# Perceived Threat of COVID-19 Contagion and Frontline Paramedics’ Agonistic Behaviour: Employing a Stressor–Strain–Outcome Perspective

**DOI:** 10.3390/ijerph17145102

**Published:** 2020-07-15

**Authors:** Fakhar Shahzad, Jianguo Du, Imran Khan, Adnan Fateh, Muhammad Shahbaz, Adnan Abbas, Muhammad Umair Wattoo

**Affiliations:** 1 School of Management, Jiangsu University, Zhenjiang 212013, China; 2 Department of Management Sciences, The Islamia University of Bahawalpur, Punjab 63100, Pakistan; dr.imran.khan@outlook.com (I.K.); umairwattoo26@yahoo.com (M.U.W.); 3 Faculty of Business and Accountancy, University of Malaya, Kuala Lumpur 50603, Malaysia; adnanfateh1234@gmail.com; 4 Lyallpur Business School, Government College University Faisalabad, Faisalabad 38000, Pakistan; shahbaz755@yahoo.com; 5 School of Economics and Management, Harbin University of Science and Technology, Harbin 150080, China; adnan.abbas001@yahoo.com

**Keywords:** COVID-19, anxiety, depression, agonistic behaviour, social support

## Abstract

Historically, infectious diseases have been the leading cause of human psychosomatic strain and death tolls. This research investigated the recent threat of COVID-19 contagion, especially its impact among frontline paramedics treating patients with COVID-19, and their perception of self-infection, which ultimately increases their agonistic behaviour. Based on the stressor–strain–outcome paradigm, a research model was proposed and investigated using survey-based data through a structured questionnaire. The results found that the perceived threat of COVID-19 contagion (emotional and cognitive threat) was positively correlated with physiological anxiety, depression, and emotional exhaustion, which led toward agonistic behaviour. Further, perceived social support was a key moderator that negatively affected the relationships between agonistic behaviour and physiological anxiety, depression, and emotional exhaustion. These findings significantly contributed to the current literature concerning COVID-19 and pandemic-related effects on human behaviour. This study also theorized the concept of human agonistic behaviour, which has key implications for future researchers.

## 1. Introduction

Since December 2019, the global health system has been fighting with the growing number of cases of COVID-19, a viral respiratory syndrome that first appeared in China and tentatively named 2019-nCoV1 or SARS-CoV-2 [1]. The World Health Organization has assessed that the rate of COVID-19 spread is expected to be very high and long-lasting [2]. As of 4 July 2020, the confirmed number of patients with COVID-19 had reached 11.108 million, causing over 525,790 mortalities worldwide [3]. The rare history and lack of vaccines to control this novel virus may also cause a high level of panic. During a panic, healthcare personnel (in this study, paramedics, defined as “a person who is trained to give emergency medical treatment of sick persons or assist medical professionals”) face not only physical challenges but also mental burdens, including psychological distress and fear [4,5]. The unexpected rise in confirmed cases has brought huge self-infection threats and physical and mental pressure to frontline paramedics [6]. Many health professionals’ worldwide have become infected and died [7]. The threat of contagion is very high due to the novel nature of COVID-19 and its exponential spread rate compared to other diseases that paramedics encounter every day. Meanwhile, the lack of availability of appropriate drugs to treat COVID-19 patients is another potential cause of the high threat from the current pandemic. This risk is bound to alter frontline paramedics’ behaviour and working conditions, and it may influence the treatment of patients. Thus, a study is needed to measure the pervasiveness of several health disorders caused by the threat of self-infection from COVID-19 among frontline paramedics treating patients with COVID-19.

Owing to the increased mortality rate associated with the virus, healthcare professionals and the public have experienced psychological concerns such as anxiety, depression, and emotional exhaustion [8]. Healthcare personnel not only tolerate too much workload but also have an extreme risk of self-infection [9]. This risk and the accompanying work environments with inadequate protection, lack of contact with family members, frustration, prejudice, and fear of getting infected further exacerbate the noted psychological health issues [10]. Furthermore, prolonged fear of illness increases individuals’ health disorders [9], leading to behavioural shifts. Similarly, a prior study posited that perceived threats and the resulting anxiety, discomfort, emotional stress, adaptation difficulties, and depression affect behavioural changes [11]. Concurrently, mass tragedies, especially those involving infectious diseases, often prompt high fear that causes enormous interruptions to individuals’ behaviour and psychological well-being [12]. In contrast with the current literature and editorial reviews, this investigation expects to lure public focus to the agonistic behaviour of frontline paramedics, which is caused by their physiological anxiety, depression, and emotional exhaustion.

Psychosocial interventions have demonstrated that stress-related ailments may affect individual behaviours. The negative consequences of stressors are especially common in humans; perhaps since we have a high degree of symbolic thinking, which may cause a constant strain response to various adverse living and working environments [13]. Based on the assessment of perceived threat, humans and other animals respond accordingly. Therefore, considering the theory of agonistic behaviour [14], this research examined the agonistic behaviour of frontline paramedics treating patients with COVID-19. Agonistic behaviour refers to “the complex of aggression, threat, appeasement, and avoidance behaviours that occur during encounters between members of the same species” [15]. Agonistic behaviour varies among species, which is integrated with a threat, aggression, and submissive interaction. These are also related to aggression in function and physiology; but not in the narrow sense of aggressive behaviour [16]. A prior study [17] extended the theory of agonistic behaviour from the biology and psychology disciplines into the context of marketing and found the impact of perceived scarcity, which increases attractiveness and leads to buying behaviour. However, one piece of knowledge is still unknown about human agonistic behaviour, and a paucity of theoretical development in measuring human agonistic behaviour motivated the authors to develop an in-depth understanding of this vital concept.

Therefore, the authors further extended the theory of agonistic behaviour from the field of animal biological sciences to human behavioural science. The authors integrated the stressor–strain–outcome (SSO) model with the theory of agonistic behaviour to examine the effects of the perceived threat of COVID-19 on human agonistic behaviour. The research contributes by developing and validating the theoretical framework using real-life scenarios—frontline paramedics treating patients with COVID-19. This informs the current transformation of studying animal agonistic behaviour to human agonistic behaviour. Specifically, this study explains that the higher the perceived threat level of COVID-19, the higher the degree of physiological anxiety, depression, and emotional exhaustion leading towards agonistic behaviour among frontline paramedics.

Another factor related to agonistic behaviour is perceived social support (PSS), which refers to individuals’ feelings of being cared for, valued, loved, and having a sense of belonging to those who are relied on [18]. Several studies have shown that, for people with anxiety and depression, a higher sense of social support may be psychologically comforting [17,18,19,20]. Of course, social support can have a salutary effect on health. Concurrently, the potential moderating effect of PSS on human agonistic behaviour has received little interest from scholars. Therefore, our study also examined the moderating role of PSS on the association between selected strain factors (physiological anxiety, depression, and emotional exhaustion) and the agonistic behaviour of frontline paramedics. In this study context, understanding human agonistic behaviour will help to determine specific characteristics and potential mechanisms of human aggression and violence in a variety of contexts.

## 2. Theoretical Support and Conceptualisation

### 2.1. Theory of Agonistic Behaviour

Agonistic behaviour is also known as agonism—survivalist animal behaviour, including defence, avoidance, and aggression. The term agonistic behaviour was first used to describe animal fighting behaviour [21]. It is an adaptive behaviour resulting from conflicts within the same species members [14]. While there is no commonly accepted definition of human agonism, it has usually been defined as the act of triggering psychological or physical harm to other persons or in the destruction of property [15]. Moreover, it is further defined as ‘the individual’s aggressive verbal and physical tendencies and aggressive attitudes’ [22]. Agonistic behaviour can serve as a tool for distinct antisocial, constructive activities, and destructive acts. In both human and non-humans, agonistic behaviour is significantly influenced by the general principles of operant and classical conditioning learning and social modelling [17]. The biologist who favoured this concept recognised that behavioural stimuli and underlying feelings and approaches are frequently the same; and actual behaviour is dependent on other factors, especially distance to the stimulus [23]. Moreover, the term ‘agonistic’ introduces that the differences between aggressive and agonistic behaviours have been blurred, and these two labels are often used interchangeably in the literature. In humans, aggression is repeatedly related to living conditions [17].

Behaviour also depends on the level of awareness among group members when stressful events occur in a social environment because individuals are susceptible to behavioural signals [24]. One primary reaction during the pandemic is fear of contagion. Humans react like other animals because they have a similar defence system against ecological threats [25]. Negative emotions brought about by threats can be contagious, and fear makes threats more imminent [26,27]. Behaviour has, in part, a genetic basis, which generally is learned in a social context. Several factors can cause positive and negative behavioural change. Previous literature discussed the change in agonistic behaviour of animals species rather than the human species. This study thus empirically investigated agonistic behaviour in humans and assessed the effect of the perceived threat of COVID-19 on agonistic behaviour by employing the SSO model. This study will make a significant contribution to the existing theory of agonistic behaviour by elucidating how to measure human psychological cognition and behaviour.

### 2.2. SSO Perspective

Our framework is based on the SSO model because we examined the influence of the perceived threat of COVID-19 (a stressor) on agonistic behaviour [28]. This model divulges how stressors become prominent in individuals’ lives, indicating that the stressor source has a direct influence on the strain, which later contributes to outcome variables. Stressors are environmental stimuli that individuals experience and transmit stress. Strain and outcomes are an individual’s personal emotional, and behavioural responses to stressors [13]. Summing up, the SSO model considers that strain is the result of sensing stressors and the antecedent of the outcome variable. In the past, SSO models have been used to comprehend stress in the workplace and behavioural change as an outcome variable [29,30,31,32]. However, in the context of measuring agonistic behaviour among humans, the implementation of the SSO model has not been sufficiently investigated.

With the rapid rise in COVID-19 cases, the severe threat to medical staff is imminent, which increases their physiological and psychosomatic strain [33]. In addition, the availability of equipment and pandemic control preparedness may have a moral effect on medical personnel [34]. However, the threat of getting sick from COVID-19 persists, which also puts stress on paramedical personnel. This stress further affects the psychosomatic state of frontline paramedics and increases their agonistic behaviour. Recent studies have also confirmed that the perceived fear of COVID-19 contagion affects individuals’ psychological distress [12,34,35].

Since outcome factors interact with psychological responses and perceived stressors, the current research model included three valuable and practical individual strains. The first is physiological anxiety—“a level and nature of anxiety, including physiological worry/oversensitivity, social concerns and concentration” [36]. The second is depression—“a mental illness with physiological and psychological consequences, including sluggishness, diminished interest and pleasure, and disturbances in sleep and appetite” [37]. The third is emotional exhaustion—“the extent to which employees feel drained and overwhelmed by their work” [31].

In this study, agonistic behaviour—“adaptive acts which arise out of conflicts between two members of the same species”—was our dependent variable [15]. In prior literature, it was mostly used interchangeably with aggressive behaviour. Few scholars have discussed human agonistic behaviour, particularly in the field of marketing and customers’ buying behaviour [15,17]. However, there is no empirical evidence concerning the impact of the perceived threat of COVID-19 or any other pandemic-related fears from the perspective of the SSO model. This motivated the authors to investigate the possible consequences of human agonistic behaviour. The SSO model can be an effective way because it emphasises the positive effect of the environmental stimulus on the internal and external behaviour of frontline paramedics treating patients with COVID-19. Moreover, the sequential process of the SSO model has been used to test the theoretical avowals made in this study, which includes how perceived threat of COVID-19 affect the agonistic behaviour of frontline paramedics by creating physiological anxiety, depression, and emotional exhaustion.

## 3. Research Model and Hypotheses Development

### 3.1. Perceived Threat of COVID-19

In this section, we will discuss how threats and risks may be perceived and responded to by people during a pandemic and its aftermath; specifically, fear causes individuals to change their behaviour. Intense fear produces the greatest behavioural changes when people experience physical and psychosomatic disorders such as anxiety, depression, and emotional exhaustion [38,39,40]; whereas intense fear can lead to aggressive and defensive responses [26]. Therefore, we adapted the previous Brief Illness Perception Questionnaire (BIPQ) [41] to determine the level of perceived threat among frontline paramedics treating patients with COVID-19. The concept of illness perception is related to how a person perceives the illness as well as the cognitive structuring of the status of being ill. The model recommends that situational stimuli can produce cognitive and emotional representations of health threats or illness [41]. In other terms, illness perception is the cognitive and emotional representations of patients’ viewpoints about the disease [42].

This cognitive and emotional model also includes beliefs about the treatment and control of the situation. The emotional and cognitive interpretation and evaluation about the perception of illness are the determinants of their behavioural reactions, which is shaped by individuals’ experiences, knowledge levels, and mental strain [43]. Therefore, per prior directions [43], we divided and validated the scale into two parts based on the emotional and cognitive perception of the threat of illness from COVID-19.

First, emotional threat is a psychological disorder characterised by uncontrollable and irrational fears, extreme hostility, or persistent anxiety. It identifies the illness consequences and concern that affect individuals’ emotions and create anxiety and depression, making them angry, scared, and exhausted [41,44,45]. However, it is not the amount of emotions but rather the interpretation of emotional states that is essential for determining an individual’s degree of psychological disorder [46]. They confirmed a relationship between the level of distress intolerance, anxiety, and bulimic behaviour in a non-clinical setting [46]. Second, cognitive threat refers to the identification of an illness threat from a particular disease, understanding its expected effects, and lacking personal control over the situation [44]. It may also contribute to the creation of anxiety disorders and psychological distress, which ultimately leads to behavioural change [41,43,47].

Fear of illness is inextricably linked with depression and anxiety [48]. Per Chinese scholars, a parallel epidemic of depression, anxiety, and emotional exhaustion is triggered by the COVID-19 pandemic [4,49]. In addition, recent studies posited that the pandemic had provoked widespread psychological issues, such as fear, anxiety, and depression, among countries with a high prevalence of viral infections [50,51]. Similarly, we assumed that perceived emotional and cognitive threat concerning COVID-19 would create anxiety, depression, and emotional exhaustion among the paramedics treating patients with COVID-19, which would ultimately lead to their agonistic behaviour (i.e., outcome). Thus, we hypothesised the following:

**H1a:**  *Perceived emotional threat will be positively related to physiological anxiety*.

**H1b:**  *Perceived emotional threat will be positively related to depression*.

**H1c:**  *Perceived emotional threat will be positively related to emotional exhaustion*.

**H2a:**  *Perceived cognitive threat will be positively related to physiological anxiety*.

**H2b:**  *Perceived cognitive threat will be positively related to depression*.

**H2c:**  *Perceived cognitive threat will be positively related to emotional exhaustion*.

### 3.2. Physiological Anxiety

Anxiety disorders are often caused by stressful life events [13]. Anxiety is defined as “an emotion characterized by feelings of tension, worried thoughts and physical changes like increased blood pressure” [52]. Anxiety is also the cause and effect of many psychosomatic diseases and plays a role in the development of emotional psychosis [16]. Prior literature described the possible role of stress and fear of sickness in the causation of submissive behaviour owing to anxiety [11,53,54]. How long the novel coronavirus will persist and how it will continue to influence the psychological well-being of healthcare staff is unknown. This psychological influence may lead to adverse behavioural change [55]. Thus, we posited that physiological anxiety will increase extensively if the pandemic persists, which ultimately will increase frontline paramedics’ agonistic behaviour. Thus, we also proposed the following hypothesis:

**H3:**  *Physiological anxiety will be positively related to agonistic behaviour*.

### 3.3. Depression

Depression refers to a ‘psychological state of low mood and aversion to activity that can affect a person’s thoughts, behaviour, motivation, feelings, and sense of well-being’ [56]. The maladaptive actions in behavioural theories have underlined the occurrence of depression. Cognitive behavioural therapy assumes that the root cause of depression is negative thinking patterns, which then lead to negative behavioural patterns [57]. People with depression have extremely negative views about themselves and the world. It is believed that long-lasting emotional stress is the pathogenic factor leading to the development of individual depression that leads to behavioural disorders [16,58]. Generally, during the early stages of a pandemic, people have little information about treatment and mortality, which exacerbates people’s fear of infection, leading toward behavioural consequences [59]. Consistently, depression rates are higher during the COVID-19 pandemic as compared to before [6]. Like anxiety, we posited that depression would increase the agonistic behaviours of frontline paramedics:

**H4:**  *Depression will be positively related to agonistic behaviour*.

### 3.4. Emotional Exhaustion

Emotional exhaustion is a stress-related social issue that may affect individuals’ working behaviour [60]. It describes ‘feelings of being emotionally overextended’ [61]. Consequences of emotional exhaustion can lead to behavioural disorders, a preference for remaining at home, and poor work performance [61,62]. Some studies have investigated the causes or consequences of employees’ emotional exhaustion in work-related environments [31,62,63,64,65]. Moreover, one study [63] concluded that greater levels of perceived pandemic threat could be used to anticipate increased levels of emotional exhaustion, leading to increased agonistic behaviour. Given that the increased threat of the COVID-19 pandemic predicts increased emotional exhaustion, it is reasonable to suggest that increased emotional exhaustion will contribute to exacerbated agonistic behaviour among frontline paramedics treating patients with COVID-19. Like anxiety and depression, we hypothesised the following:

**H5:**  *Emotional exhaustion will be positively related to agonistic behaviour*.

### 3.5. The Moderating Role of Perceived Social Support

Social support is defined as “social interactions or relationships that provide practical assistance to individuals or embedding individuals into a social system that is considered to provide love, care, or attachment to a valuable social group” [24]. Simply, social support refers to all kinds of support that individuals obtain from others. Social support is divided into actually received support and perceived support. Although the received social support includes the assistance already provided, PSS is a faith that these assisting behaviours will occur when needed in the future [66]. Increased social support is coupled with better psychological outcomes, and PSS (rather than actual social support) seems to indicate healthier psychological behaviours during times of stress [26]. Moreover, PSS was identified in the SARS outbreak and organisational behaviour literature as adversely associated with burnout [67]. Therefore, PSS was selected as the focus of this research.

Various aspects of sociocultural background influence the degree and speed of behavioural change. Social norms influence employees’ behaviours, what they think about others’ actions, and what they agree or disagree with at the workplace [68]. In addition, many studies have confirmed the relationship between decreased adolescent social support and increased aggression [69,70,71,72,73]. Moreover, greater levels of perceived pandemic threat predict resulted in increased levels of psychological strain, whereas greater social support predicts a decreased effect of psychological strain on behaviour disorders [63]. Increased PSS also protects individuals with high levels of victimisation from increased health disorders such as depression, anxiety, emotional exhaustion [19,69]. The moderating role of PSS using the stress-buffering model was also a significant contributor to depressive symptoms among Chinese nurses [20]. Nonetheless, few studies have explored the impact of PSS on the relationship between COVID-19-related stress and psychological well-being [74,75].

Consequently, we posited that PSS would buffer or moderate the relationship between strain (physiological anxiety, depression, emotional exhaustion) and outcome (agonistic behaviour). Specifically, we hypothesised the following:

**H6a:**  *PSS will moderate the positive association between physiological anxiety and agonistic behaviour; i.e., a rise in PSS will decrease the relationship strength between physiological anxiety and agonistic behaviour*.

**H6b:**  *PSS will moderate the positive association between depression and agonistic behaviour; i.e., a rise in PSS will decrease the relationship strength between depression and agonistic behaviour*.

**H6c:**  *PSS will moderate the positive association between emotional exhaustion and agonistic behaviour; i.e., a rise in PSS will decrease the relationship strength between emotional exhaustion and agonistic behaviour*.

**Figure 1:**
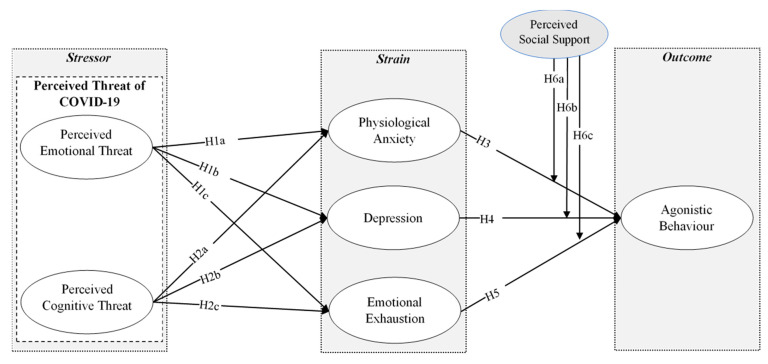
Proposed research model.

The proposed model of this study is shown in Figure 1.

## 4. Material and Method

### 4.1. Context Selection

The threat of COVID-19 initially started after the first case was reported in China. Regardless of common health issues, developing countries are still in the initial phases of tackling this uncertain situation. The COVID-19 pandemic was first verified to have arrived in Pakistan in February 2020 [76] and grew to 69,496 confirmed cases by 31 May 2020 [77]. Paramedics, working in isolation wards, fever clinics, intensive care units and other related departments with an increased workload and risk of infection. In this study, the targeted population encompassed paramedics treating patients with COVID-19 in Pakistan who completed a survey.

### 4.2. Construct Operationalisation

We adapted the survey items (See Appendix A) for all constructs from prior literature and refined them to fit the context of this research before final data collection. However, in the preliminary analysis, an item from PSS (item number 6) was excluded owing to low factor loadings and to authenticate the results [78]. Moreover, to confirm the content validity of the proposed survey, a team composed of one professor and four scholars were requested to verify the wording and face validity of the survey questionnaire. The approved questionnaire was then distributed for data collection.

#### 4.2.1. Perceived Threat of COVID-19

In this study, the Brief Illness Perception Questionnaire (BIPQ) was adapted [41] to measure the perceived threat of COVID-19 (0 to 10 scale) among frontline paramedics treating patients during the current pandemic. The initial eight-item questionnaire was divided into two categories as per prior directions [43]: perceived emotional threat and perceived cognitive threat. A sample item for the perceived emotional threat was, “How much does your threat of illness from COVID-19 affect you emotionally”? A sample item for the perceived cognitive threat was, “How well do you feel you understand COVID-19”?

#### 4.2.2. Physiological Anxiety (PA)

Physiological anxiety was measured using 11 items (7-point Likert scale) [36], which were obtained from an earlier measure [53]. A sample item was “I cannot concentrate on a task or job without irrelevant thoughts intruding”.

#### 4.2.3. Depression (DP)

Depression was measured using 19 items (7-point Likert scale) adapted from an earlier study [79]. A sample item was, “How often was this happen during the past 10 days; you were bothered by things that usually do not bother you?

#### 4.2.4. Emotional Exhaustion (EE)

Emotional exhaustion was measured using 12 items (7-point Likert scale) adapted from an earlier study [31], which were obtained from an earlier measure [80]. A sample item was, “It is hard for me to relax after dealing with COVID-19 patients”.

#### 4.2.5. Perceived Social Support (PSS)

Perceived social support was assessed using 8-items (7-point Likert scale) adapted from an earlier study [81]. A sample item was, “How much do you feel that your family pays extra attention to you during a current pandemic”?

#### 4.2.6. Agonistic Behaviour (AB)

An aggression scale was adapted from an earlier study [22] as an objective gauge to assess individuals’ agonistic behaviour. We critically analysed several aggression scales; however, we found Regoeczi’s aggression scale to be the most relevant to our definition of agonistic behaviour. A 5-items scale (7-point Likert) was administered to participants. A sample item was, “How often did you feel you were too aggressive toward other people during the past 10 days”?

### 4.3. Data Collection, Sampling, and Analysis Procedure

Consistent with the focus of this study, data were gathered through a structured questionnaire only from paramedical personnel treating patients with COVID-19 in Pakistan. In the Punjab province of Pakistan, there are two separate layers of professionals that support core medical personnel in their healthcare services, namely “paramedics” and “allied health professionals”. Paramedics are registered with Punjab Medical Faculty (PMF), and Allied Health Professionals are registered with the Higher Education Commission (HEC) [82]. In this study, we have collected the data only from the frontline paramedics working in Punjab, Pakistan particularly dealing with COVID-19 patients. For this, we contacted the head of several quarantine centres and hospitals treating patients with COVID-19 around Punjab province, Pakistan. They were informed of the study purpose. All possible questions were answered to their satisfaction, but no official data were collected to assure the privacy of the respondents and the organisations. After getting verbal permission from the concerned authority, we started our data collection process.

Data collection followed the computer-assisted web interview method—a data-gathering technique in which participants complete questionnaires through an online survey link without the guidance of the interviewer [83]. The expected circulation of the survey was around 1500 using snowball sampling. A total of 372 responses were recorded between 3 March 2020 and 17 May 2020. Twenty-seven responses were omitted from final analyses because they were deemed unreliable [84]. Moreover, the same size exceeding 200 meant it was reasonable to employ structural equation modelling (SEM) [85]. Considering the length of the survey (66 questions), utilising SEM analyses was rational. Moreover, we evaluated the sample adequacy on the advice of [86], based on Cohen’s power theory. A post-hoc was applied for all exogenous indicators (significance level was set at 0.05, the effect size was 0.15, and the sample size was 345) to verify the statistical intensity of the study sample using G*power 3.1.9 (Heinrich-Heine-Universität, Düsseldorf, Germany) [87]. The results of the post-hoc test revealed that the statistical power was 0.9, much higher than the 0.8 thresholds [88]. Therefore, the final sample of 345 respondents was analysed by implementing the partial least square SEM technique in Smart-PLS v3.2.9 (Smart-PLS GmbH, Bönningstedt, Germany). For our purposes, this was more suitable than covariance-based SEM [89,90].

## 5. Results

### 5.1. Participants’ Demographics

Table 1 outlines participants’ characteristics (e.g., sex, age, and work experience): 38.6% were men, and 61.4% were women; 18.3% were aged ≤ 29 years old, 39.4% were aged 30 to 39 years, 40.6% were aged 40 to 49 years, and 1.7% were aged ≥ 50 years; and 20.9% had one to three years of work experience, 25.2% had four to six years of work experience, 26.1% had seven to nine years of work experience, and 27.8% had ≥ 10 years of work experience.

### 5.2. The Empirical Results of the Measurement Model

#### 5.2.1. Reliability and Convergent Validity

Convergence validity indicates the correlation level of several indexes in a parallel structure [78]. To verify the convergent validity of each item, Smart-PLS v3.2.9 software was used to conduct a confirmatory factor analysis. Table 2 shows the reliability and convergent validity of this study. In addition, Cronbach’s alpha of all factors ranged from 0.934 to 0.974, which was higher than the threshold value. Concerning convergent validity, this study examined the similarity between operationalisation and theory. The composite reliability (CR) was 0.947 to 0.976, and the average variance extracted (AVE) was 0.684 to 0.861.

The suggested values for Cronbach’s alpha and CR should be greater than 0.7, and AVE should be greater than 0.5; thus, the instrument was efficient and reliable [78,91] and the data could be used for further structural analysis.

#### 5.2.2. Discriminant Validity

To distinguish the extent of empirical variance among the constructs, discriminant validity evaluation has become a widely accepted assumption to analyse the relationship between potential factors [89]. In this study, we used three methods to evaluate discriminant validity. First, by associating the correlation of the factors with the square root of the AVE. Second, the survey items were checked through the cross-loading criterion to recognise the relevance. Third, discriminant validity was measured by the application of Heterotrait-Monotrait Ration (HTMT) [89,92,93].

As described in Table 3, the correlation between constructs and the square root of AVE was linked to quantify the discriminant validity of the instrument. The diagonal values in Table 3 suggest that the square root of AVE is higher than the correlation coefficients between all variables, a good indication of discriminant validity [93].

Prior studies suggested cross-loadings criteria to assess discriminant validity [91,94]. Accordingly, the loading of each item should be higher than its subsequent construct, and the item loadings are also regarded as a threshold. The calculation results of item loadings and cross-loadings (see Table 4) show that the loadings of each item are higher than the cross-loadings of other subsequent construct items. This shows that it has sufficient discriminant validity by satisfying the cross-loading criteria.

Finally, the HTMT ratio criterion was established to illustrate the insensitivity of Fornell and Larcker’s criterion and cross-loading criterion. The ratio of HTMT was close to 1, indicating the lack of discriminant validity [91]. HTMT is an estimate of factor correlation (or instead, the upper bound). To make a clear distinction between the two factors, HTMT should be less than 1 [92,95]. Therefore, we employed the HTMT ratio; the value in Table 5 shows that the highest value is 0.75, which is lower than the above threshold, indicating sufficient discriminant validity.

### 5.3. The Empirical Results of the Structural Model

**Figure 2:**
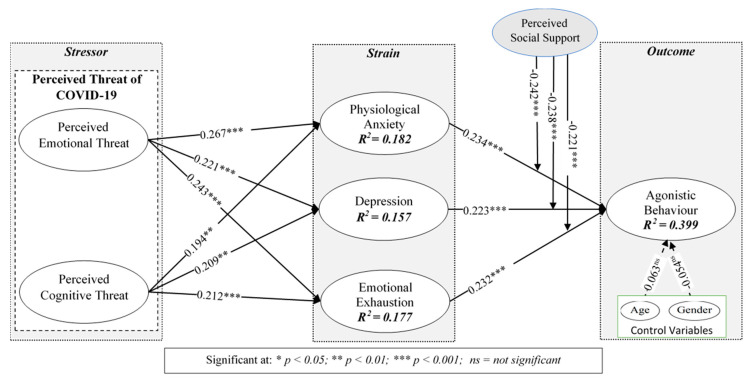
SEM results for hypotheses testing.

After examining reliability and validity, we measured the causal relationship between the factors with Smart-PLS v3.2.9 software [89,95]. Figure 2 shows the value of the path coefficient. The bootstrap technique was used to measure the significance of the structural model (2000 iterations of resampling). The expressive power of the research model is represented by the illustrative variation of its results (i.e., R^2^). The R^2^ (R-Square) value of AB was 0.399, indicating that these selected variables represented 39.9% of the variation. Moreover, the R^2^ of physiological anxiety was 0.182, indicating that the mutation rate owing to perceived emotional threat (PET) and perceived cognitive threat (PCT) was 18.2%. In addition, the R^2^ of depression was 0.157 and the R^2^ of emotional exhaustion was 0.177, indicating the active participation of perceived threat.

The SEM results in Figure 2 show that all exogenous factors are positively associated with endogenous factors. The *p*-value confirms the level of significance of the relationship between the proposed relations per the criterion [96,97]. Meanwhile, the value of Standardized Root Mean Square Residual (SRMR) is 0.042, and the value of Normed Fit Index (NFI) is 0.891, showing the good fitness of the model. In Figure 2, the SEM analysis results verify the path analysis coefficient between PET and physiological anxiety is (β = 0.267, *p* < 0.001). PET had a significant positive effect on physiological anxiety, and the beta correlation coefficient between PET and depression was significant (β = 0.221, *p* < 0.001). The findings further indicated that PET and emotional exhaustion were significantly positively correlated (β = 0.243, *p* < 0.001). Based on these statistical findings, H1a, H1b, and H1c were supported.

The beta coefficient of PCT was significant (β = 0.194, *p* < 0.01), implying that it positively impacted physiological anxiety; therefore, H2a was supported. PCT was positively correlated with depression and emotional exhaustion. PCT and depression were also significantly positively correlated (β = 0.209, *p* < 0.01), as were PCT and emotional exhaustion (β = 0.212, *p* < 0.001). Therefore, H2b and H2c were supported.

Physiological anxiety also had a considerable effect on AB (Figure 2; β = 0.234, *p* < 0.001). The coefficient values of depression and AB (β = 0.223, *p* < 0.001) and emotional exhaustion and AB (β = 0.232, *p* < 0.001) indicated that the selected strain factors (physiological anxiety, depression, and emotional exhaustion) had a substantial positive effect on the AB. Therefore, H3, H4, and H5 are were supported.

### 5.4. The Moderating Role of Perceived Social Support

**Figure 3:**
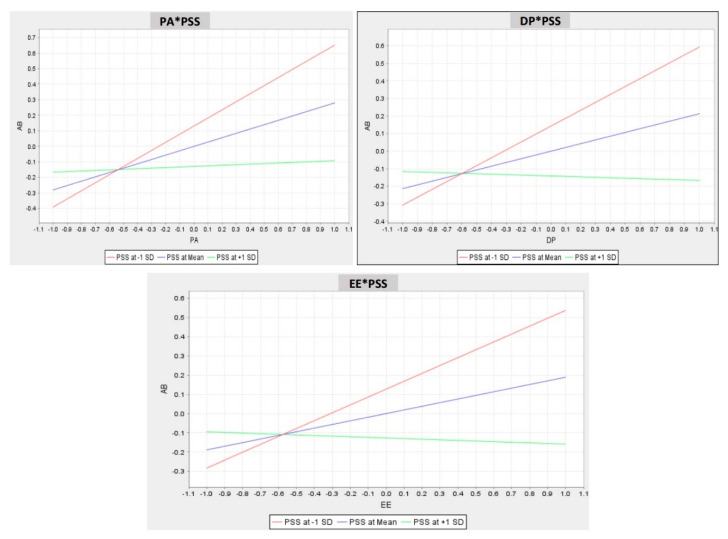
Moderating effect of PSS on the relationship of PA, DP, and EE with AB. DP = depression; EE = emotional exhaustion; PA = physiological anxiety; PSS = perceived social support.

Figure 2 shows the interaction value of the beta coefficient of PSS on the association between physiological anxiety and AB (β = −0.242, *p* < 0.001), the coefficient value of PSS on the association between depression and AB is (β = −0.238, *p* < 0.001), and the coefficient value of PSS on the relationship between emotional exhaustion and AB (β = −0.221, *p* < 0.001). PSS significantly and negatively influenced the relationships between physiological anxiety, depression, and emotional exhaustion with AB (Figure 3). Consequently, H6a, H6b, and H6c were supported. Figure 3 also illustrates the moderating effect of PSS on the relationship between physiological anxiety, depression, and emotional exhaustion with AB. In sum, per the present analyses, the proposed theoretical model was acceptable.

### 5.5. Common Method Bias and Multicollinearity

The common method bias (CMB) possibly exposes the efficacy of this study. The survey notes informed participants that there were no right or wrong answers and that their replies would remain anonymous and confidential. Moreover, Harman’s single factor test is usually used to test for the existence of CMB [98,99]. We used SPSS v26 (IBM SPSS Inc., Chicago, IL, USA) software to perform Harman’s single factor test.

The first factor accounted for 40.9% of the variation. In social science literature, a value below 50% is the threshold of the CMB [98,100,101]. Concurrently, the inner variance inflation factor (VIF) was also used to evaluate the CMB problem. According to Kock (2015), inner-VIF should be less than 3.3. We discovered that the value varied between 1.09 to 2.01; thus, CMB was not a problem in this study.

The values of outer-VIF were used for multicollinearity assessment of the survey items. The literature shows that if the VIF value of a study is lower than 10, multicollinearity may not be a problem [102,103,104]. The highest value of VIF was 5.93; thus, there was no severe multicollinearity problem. In sum, the proposed model did not have CMB or multicollinearity problems, indicating that the structural model measured significant differences between the constructs.

## 6. Discussion

The global understanding of disease transmission and management has improved during the several pandemics in history. However, COVID-19 has limited global health authorities’ abilities. As previous studies disclosed, working directly with patients will increase individuals’ fear of getting sick and uncertainty about pandemic contagion [63,105], which we called perceived threat of COVID-19 in this study. Therefore, we investigated the impact of perceived COVID-19 threat in forecasting greater levels of physiological anxiety, depression, and emotional exhaustion among frontline paramedics, which may boost their agonistic behaviour. Another objective was to examine the moderating influence of PSS in reducing the adverse consequences of physiological anxiety, depression, and emotional exhaustion on agonistic behaviour owing to the perceived threat of COVID-19. The BIPQ [41] was used to measure the perceived threat of COVID-19, which was divided into two constructs: perceived emotional threat and perceived cognitive threat. SEM was applied to the data to test the research model under the podium of the SSO framework.

The results revealed that frontline paramedics in the isolation wards did not think that they were exempt from the peril, which was associated with increased psychological distress. Moreover, paramedics worried about the inadequacy of protective measures and vigilance taken by the health department. Paramedics’ perception of risk contributed to their psychological morbidity and irregular behaviour. Based on the empirical results, we postulated that an increased perceived threat of COVID-19 would increase the level of paramedics’ physiological anxiety and depression, which would ultimately increase their agonistic behaviour. A causal link between the perceived threat of COVID-19 and psychological distress was found. After working in isolation for a considerable period, paramedics reported emotional exhaustion. Treating patients with COVID-19 had become routine, and they were inured to being around death almost every day. However, they also experienced substantial stress owing to the fear of getting ill during the pandemic. The cognitive and emotional threat from COVID-19 was positively associated with increased emotional exhaustion at work, which was associated with paramedics’ behavioural change.

Moreover, the results showed that PSS reduced the effect of anxiety, depression, and emotional exhaustion on agonistic behaviour. PSS is helpful as friends or family members provide social support and express empathy. With the increase in the number of cases of COVID-19 infection around the globe, frontline paramedics are required to wear protective masks, protective clothing, and treat many patients with COVID-19, which may cause added stress. However, PSS can help reduce this stress by reducing the perception of the threat of stressful events and the physiological response and inappropriate behaviour that can result from stress. These results are also supported by prior studies [75,106]. Positive social feedback should thus be provided to frontline paramedics in times of uncertainty to offset potential agonistic behaviour.

## 7. Implications, Limitations, and Research Directions

### 7.1. Theoretical Implications

First, this research offers a more account of the theory of agonistic behaviour from the field of animal biological sciences to human behavioural science. The authors integrated the SSO model with the theory of agonistic behaviour to examine the effects of the perceived threat of COVID-19 on human agonistic behaviour. This empirical investigation elucidated human behaviour research. Second, by using the SSO model, this study tested several theoretical-based relationships between the perceived threat of COVID-19 and human agonistic behaviour. Most of the recent studies concerning COVID-19 discussed the consequences and adverse effect on patients’ health, daily life, economy, and education [4,55,107,108,109,110]; however, this study mainly concentrated on the perceived threat of COVID-19 among frontline paramedics, and how it influenced their psychological strain and increased their agonistic behaviour. Therefore, the authors hope that this model can be further extended and used as an ideal platform for future work in a similar context. Third, this study further divided the BIPQ into two major parts—emotional and cognitive threats—and empirically tested it during the current pandemic situation. This significantly contributes to validating the existing scales and can be used in future research.

### 7.2. Practical Implications

This study also provides some useful insights for practice. First, the findings significantly highlighted the risk of infection that frontline paramedics face, which may cause several mental health problems such as anxiety and depression. Health organisations should implement full security measures to protect this at-risk population to mitigate the threat of COVID-19. Second, the results emphasised the need for healthcare managers to understand the magnitude and sources of psychosocial stress faced by frontline paramedics. Providing adequate protection and facilities, communicating effectively, creating transparent guidelines, and implementing appropriate feedback mechanisms for healthcare personnel are essential to reduce the strain in the current pandemic situation. Third, this study highlights the significant role of PSS in reducing the effect of psychological strain on agonistic behaviour. Concerning stress management, it is also essential to strengthen social support in the workplace. For frontline paramedics with severe psychological strain, it is necessary to identify high-risk groups early, and provide counselling, social support, and stress management to mitigate negative behavioural change.

### 7.3. Limitations and Research Directions

Some limitations need to be addressed while discussing the outcomes of the current study. First, a cross-sectional design was employed, and the agonistic behaviour of paramedics was measured during the current pandemic. Future scholars should employ a multimethod or longitudinal design by comparing the results obtained during and after the COVID-19 pandemic. Second, this study did not examine sex and age differences. The level of threat may not be the same between female and male paramedics. Similarly, those in different age groups will respond to strain differently and may display agonistic behaviour in diverse ways. Therefore, multigroup analyses should examine any possible sex or age differences. Third, the strain factors discussed in this study are not limited to these particular factors; future researchers could extend the model using several other factors such as scepticism, sadism, and poor sleep quality, which may impact human agonistic behaviour. Organisational and government support can also be used as a moderating factor in addition to PSS. Finally, future researcher should continuously validate the scale used in the current study.

## 8. Conclusions

Our study concludes that the effect of perceived COVID-19 threat on predicting greater levels of physiological anxiety, depression, and emotional exhaustion among frontline healthcare paramedics may contribute to their agonistic behaviour. Moreover, we have concluded the moderating role of PSS in decreasing the adverse effect of physiological anxiety, depression, and emotional exhaustion on agonistic behaviour due to the perceived threat of COVID-19. Our study provides understanding about human agonistic behaviour will help to identify precise characteristics and probable mechanisms of human aggression and violence in several contexts, which will contribute to the implementation of conflict management practices in the workplace.

## Figures and Tables

**Table 1:** Participants profile.

Category		Frequency	% Age
Sex	Men	133	38.6
	Women	212	61.4
	Total	345	100.0
Age	20 to 29 years	63	18.3
	30 to 39 years	136	39.4
	40 to 49 years	140	40.6
	Over 50 years	06	1.7
	Total	345	100.0
Work experience	1 to 3 years	72	20.9
	4 to 6 years	87	25.2
	7 to 9 years	90	26.1
	Above 10 years	96	27.8
	Total	345	100.0

**Table 2:** Reliability and convergent validity.

Constructs	Cronbach’s Alpha	rho_A	CR	AVE
AB	0.945	0.946	0.958	0.819
DP	0.974	0.975	0.976	0.684
EE	0.961	0.962	0.966	0.703
PA	0.967	0.968	0.970	0.749
PCT	0.946	0.947	0.961	0.861
PET	0.936	0.939	0.955	0.840
PSS	0.934	0.935	0.947	0.718

AB = agonistic behaviour; DP = depression; EE = emotional exhaustion; PA = physiological anxiety; PCT = perceived cognitive threat; PET = perceived emotional threat; PSS = perceived social support. CR = composite reliability; AVE = average variance extracted.

**Table 3:** Fornell-Larcker criterion.

Constructs	AB	DP	EE	PA	PCT	PET	PSS
AB	0.905						
DP	0.531	0.827					
EE	0.539	0.639	0.838				
PA	0.513	0.611	0.560	0.866			
PCT	0.333	0.364	0.384	0.382	0.928		
PET	0.364	0.368	0.393	0.404	0.705	0.917	
PSS	−0.254	−0.178	−0.275	−0.104	0.014	−0.032	0.847

Note: Pearson correlations are shown below the diagonals. *p* < 0.05. AB = agonistic behaviour; DP = depression; EE = emotional exhaustion; PA = physiological anxiety; PCT = perceived cognitive threat; PET = perceived emotional threat; PSS = perceived social support.

**Table 4:** Cross-loadings criterion.

Items	AB	DP	EE	PA	PCT	PET	PSS
AB1	0.899	0.480	0.458	0.464	0.293	0.312	−0.215
AB2	0.921	0.491	0.561	0.473	0.329	0.331	−0.245
AB3	0.926	0.476	0.532	0.474	0.297	0.321	−0.233
AB4	0.914	0.476	0.453	0.459	0.307	0.355	−0.230
AB5	0.863	0.480	0.424	0.450	0.276	0.330	−0.222
DP1	0.409	0.775	0.453	0.544	0.260	0.268	−0.090
DP2	0.444	0.857	0.508	0.556	0.268	0.293	−0.110
DP3	0.435	0.843	0.535	0.514	0.266	0.252	−0.149
DP4	0.464	0.862	0.503	0.543	0.326	0.328	−0.114
DP5	0.486	0.843	0.551	0.552	0.338	0.326	−0.180
DP6	0.454	0.858	0.506	0.544	0.337	0.350	−0.143
DP7	0.438	0.825	0.506	0.527	0.261	0.275	−0.147
DP8	0.460	0.816	0.498	0.523	0.322	0.316	−0.140
DP9	0.473	0.845	0.557	0.504	0.352	0.360	−0.184
DP10	0.482	0.776	0.540	0.459	0.317	0.305	−0.211
DP11	0.469	0.804	0.579	0.467	0.294	0.328	−0.137
DP12	0.416	0.837	0.559	0.478	0.332	0.315	−0.137
DP13	0.410	0.855	0.536	0.511	0.290	0.256	−0.115
DP14	0.369	0.786	0.499	0.426	0.239	0.233	−0.071
DP15	0.434	0.814	0.548	0.465	0.263	0.287	−0.172
DP16	0.410	0.846	0.549	0.483	0.336	0.334	−0.178
DP17	0.415	0.857	0.526	0.510	0.293	0.315	−0.170
DP18	0.436	0.818	0.562	0.476	0.335	0.342	−0.165
DP19	0.408	0.790	0.519	0.504	0.259	0.256	−0.154
EE1	0.432	0.615	0.786	0.469	0.281	0.310	−0.269
EE2	0.447	0.572	0.791	0.486	0.258	0.305	−0.287
EE3	0.422	0.555	0.842	0.468	0.306	0.309	−0.250
EE4	0.450	0.521	0.861	0.484	0.307	0.306	−0.254
EE5	0.429	0.557	0.854	0.477	0.354	0.319	−0.213
EE6	0.498	0.583	0.870	0.474	0.356	0.362	−0.275
EE7	0.451	0.566	0.882	0.464	0.348	0.353	−0.243
EE8	0.420	0.498	0.831	0.405	0.334	0.334	−0.182
EE9	0.471	0.558	0.902	0.452	0.338	0.362	−0.273
EE10	0.461	0.511	0.833	0.500	0.346	0.385	−0.218
EE11	0.484	0.456	0.816	0.508	0.310	0.307	−0.148
EE12	0.450	0.444	0.785	0.442	0.309	0.286	−0.158
PA1	0.425	0.510	0.507	0.836	0.362	0.348	−0.126
PA2	0.413	0.537	0.500	0.847	0.343	0.332	−0.082
PA3	0.423	0.497	0.448	0.862	0.322	0.366	−0.079
PA4	0.487	0.571	0.493	0.881	0.347	0.369	−0.111
PA5	0.497	0.544	0.484	0.886	0.367	0.379	−0.122
PA6	0.420	0.503	0.497	0.880	0.356	0.387	−0.064
PA7	0.443	0.575	0.485	0.887	0.362	0.361	−0.030
PA8	0.456	0.510	0.483	0.885	0.326	0.350	−0.048
PA9	0.436	0.534	0.496	0.842	0.296	0.336	−0.111
PA10	0.456	0.518	0.479	0.866	0.278	0.318	−0.070
PA11	0.418	0.513	0.458	0.850	0.271	0.290	−0.147
PCT1	0.320	0.361	0.334	0.359	0.935	0.664	0.025
PCT2	0.306	0.314	0.355	0.327	0.928	0.690	−0.005
PCT3	0.322	0.320	0.368	0.357	0.923	0.601	−0.013
PCT4	0.287	0.356	0.366	0.374	0.927	0.663	0.043
PET1	0.337	0.324	0.356	0.350	0.632	0.931	−0.043
PET2	0.365	0.361	0.361	0.373	0.644	0.939	−0.062
PET3	0.313	0.348	0.388	0.391	0.631	0.932	−0.057
PET4	0.320	0.314	0.333	0.365	0.681	0.861	0.051
PSS1	−0.206	−0.130	−0.245	−0.097	−0.003	−0.026	0.909
PSS2	−0.222	−0.109	−0.237	−0.086	−0.032	−0.083	0.755
PSS3	−0.207	−0.191	−0.255	−0.090	0.015	−0.009	0.860
PSS4	−0.211	−0.176	−0.244	−0.082	0.006	−0.041	0.853
PSS5	−0.194	−0.128	−0.197	−0.081	0.039	−0.019	0.839
PSS7	−0.219	−0.157	−0.232	−0.103	0.064	0.005	0.856
PSS8	−0.237	−0.161	−0.220	−0.073	−0.003	−0.016	0.852

AB = agonistic behaviour; DP = depression; EE = emotional exhaustion; PA = physiological anxiety; PCT = perceived cognitive threat; PET = perceived emotional threat; PSS = perceived social support.

**Table 5:** HTMT ratio criterion.

Constructs	AB	DP	EE	PA	PCT	PET	PSS
AB							
DP	0.552						
EE	0.563	0.661					
PA	0.536	0.628	0.581				
PCT	0.351	0.376	0.401	0.398			
PET	0.388	0.382	0.413	0.423	0.751		
PSS	0.269	0.184	0.290	0.109	0.041	0.065	

AB = agonistic behaviour; DP = depression; EE = emotional exhaustion; PA = physiological anxiety; PCT = perceived cognitive threat; PET = perceived emotional threat; PSS = perceived social support.

## Appendix A: Survey Items

**Perceived Emotional Threat**
“How much does COVID-19 affect your life?”
“How much do you feel symptoms COVID-19 contagion?”
“How concerned are you about COVID-19 contagion?”
“How much does your threat of illness from COVID-19 affect you emotionally? (e.g., does it make you angry, scared, upset or depressed)”
**Perceived Cognitive Threat**
“How long do you think COVID-19 will continue?”
“How much control do you feel over COVID-19 contagion?”
“How much do you think that current treatment is helpful from the recovery of COVID-19 contagion?”
“How well do you feel you understand COVID-19?”
**Physiological Anxiety**
“I picture some future misfortune.”
“I cannot get some thoughts out of my mind.”
“I abide on mistakes that I have made.”
“I think about possible misfortunes to my loved ones.”
“I cannot concentrate on a task or job without irrelevant thoughts intruding.”
“I keep trying to avoid uncomfortable thoughts.”
“I cannot get some pictures or images out of my mind.”
“I imagine myself appearing foolish with a person whose opinion of me is important.”
“I am concerned that others might not think well of me.”
“I have to be careful not to let my real feelings show.”
“I have an uneasy feeling.”
**Depression**
How often was this happen during the past 10 days:
“you were bothered by things that usually do not bother you?”
“you did not feel like eating, and your passion was poor?”
“you felt that you could not shake the blues, even with help from family and friends?”
“you felt that you were just as good as other people?”
“you had trouble keeping your mind on what you were doing?”
“you felt depressed?”
“you felt that you were too tired to do things?”
“you felt hopeful about the future?”
“you thought your life had been a failure?”
“you felt fearful?”
“you were happy?”
“you talked less than usual?”
“you felt lonely?”
“people were unfriendly to you?”
“you enjoyed life?”
“you felt sad?”
“you felt that people disliked you?”
“it was hard to get started doing things?”
“you felt life was not worth living?”
**Emotional Exhaustion**
“It is hard for me to relax after dealing with COVID-19 patients.”
“If others speak to me, I will sometimes give an errant reply.”
“I mostly feel annoyed while dealing with COVID-19 patients.”
“I sometimes act aggressively, although I do not want to do so. “
“I feel irritable after dealing with COVID-19 patients for hours.”
“I feel emotionally drained sometimes.”
“I feel used up at the end of my work.”
“I feel fatigued when I get up in the morning and being confronted with news of COVID-19 patients.”
“I feel burned out from dealing with COVID-19 patients. “
“I feel frustrated after work.”
“I fell, I am working with COVID-19 patients for too long.”
“Dealing with COVID-19 patients puts too much stress on me.”
**Perceived Social Support**
“How much do you feel that adults care about you?”
“How much do you feel that your employer care about you?”
“How much do you feel that your parents care about you?”
“How much do you feel that your friends care about you?”
“How much do you feel that people in your family understand you?”
“How much do you feel that you want to leave home?” (deleted)
“How much do you feel that you and your family have fun together during a current pandemic?”
“How much do you feel that your family pays extra attention to you during a current pandemic?”
**Agonistic Behaviour**
“How often did you feel, you were too aggressive toward other people during the past 10 days?”
“How often did you feel, you influence other people too much to get what you want during the past 10 days?”
“How often did you feel, not at all aggressive-aggressive during the past 10 days?”
“How often did you feel, you like people to be afraid of you during the past 10 days?”
“How often did you feel, you try to get into a position of authority during the past 10 days?”
